# Kaposi Varicelliform Eruption Complicated by Steroids: A Case Report and Review of Literature

**DOI:** 10.15388/Amed.2025.32.2.4

**Published:** 2025-12-30

**Authors:** Aniket Goswami, Shikha Verma, Chingshubam Bikash, Pranjal Kalita

**Affiliations:** 1 Department of Dermatology, North Eastern Indira Gandhi Regional Institute of Health and Medical Sciences, Shillong, India; 2 Department of Dermatology, North Eastern Indira Gandhi Regional Institute of Health and Medical Sciences, Shillong, India; 3 Department of Dermatology, North Eastern Indira Gandhi Regional Institute of Health and Medical Sciences, Shillong, India; 4 Department of Pathology, North Eastern Indira Gandhi Regional Institute of Health and Medical Sciences, Shillong, India

**Keywords:** kaposi varicelliform eruption, acyclovir, tzanck smear, steroid, eczema herpeticum, Kapoši variceliforminė erupcija, acikloviras, Tzanck tepinėlis, steroidai, herpetinė egzema

## Abstract

Kaposi varicelliform eruption (KVE) is a severe disseminated cutaneous infection mainly caused by herpes simplex virus (HSV-1 or HSV-2). Herein, we report a case of KVE which was misdiagnosed elsewhere as a case of toxic epidermal necrolysis and received systemic steroids, following which, the patient’s condition deteriorated. After being diagnosed as a case of KVE with the aid of tzanck smear, the patient was initiated on acyclovir and responded dramatically to the treatment. This case underscores the critical value of simple bedside tests, such as the tzanck smear, for rapid diagnosis of KVE and the timely initiation of treatment, facilitating prompt recovery. Additionally, it highlights the importance of histopathological evaluation in ambiguous cases so that to prevent mismanagement and ensure accurate diagnosis and the appropriate care.

## Abstract (it)

Kapoši variceliforminė erupcija (KVE) yra sunki išplitusi odos infekcija, kurią dažniausiai sukelia paprastosios pūslelinės virusas (HSV-1 arba HSV-2). Pateikiame pranešimą apie KVE atvejį, kuris kitur buvo klaidingai diagnozuotas kaip toksinės epidermio nekrolizės atvejis ir pacientui buvo skirti sisteminiai steroidai, po kurių jo būklė pablogėjo. Diagnozavus KVE atvejį pagal Tzanck tepinėlį, pacientui buvo pradėtas taikyti acikloviras ir jis labai gerai reagavo į gydymą. Šis atvejis pabrėžia, kad labai svarbu atlikti paprastus tyrimus prie lovos, pavyzdžiui, Tzanck tepinėlio tyrimą, siekiant greitai diagnozuoti KVE ir laiku pradėti gydymą, kad būtų galima kuo greičiau pasveikti. Be to, reikia pabrėžti histopatologinio įvertinimo svarbą dviprasmiškais atvejais, kad būtų išvengta netinkamo gydymo ir užtikrinta tiksli diagnozė bei tinkama priežiūra.

## Introduction

*Kaposi Varicelliform Eruption* (KVE), also known as *eczema herpeticum*, is a rare but potentially catastrophic skin infection, primarily caused by herpes simplex virus (HSV-1 or HSV-2), though other viruses like coxsackie A16 and varicella-zoster may also be implicated. While some experts reserve the term *eczema herpeticum* for HSV-specific cases, KVE is used more broadly to encompass infections caused by other viruses. Initially described by Moriz Kaposi in 1887, KVE is typically associated with pre-existing cutaneous conditions like atopic dermatitis, pemphigus foliaceus, mycosis fungoides, psoriasis, and Hailey-Hailey disease. Its occurrence has also been associated with burns and various inflammatory skin disorders, reflecting its diverse etiologies. Immunosuppressive therapies, including corticosteroids, have been linked to KVE risk [1–2].

## Case Presentation

An eight-year-old boy presented with a ten-day history of painful fluid-filled lesions over the whole body starting from the face and bilateral upper and lower extremities, followed by rapid progression overnight involving the trunk and perineal region. Two days after the onset of the rash, he developed fever which would not subside on antipyretics and was associated with redness of the eyes and productive cough. His parents gave history of a similar self-limiting illness among several of his peers at school in the past week. However, they denied history of any new medication or any herbal remedies prior to the onset of his illness. They also denied history of any antecedent cutaneous conditions or symptoms suggestive of atopy. Before arriving at the current facility, the patient was treated provisionally at a local medical centre under the assumption of toxic epidermal necrolysis and received multiple courses of intravenous hydrocortisone, which reportedly exacerbated his condition. Due to the worsening of his symptoms, he was referred to a higher-level facility with a paediatric intensive care unit. The boy’s vaccination history was up-to-date per the national immunization schedule. He was a kindergarten student performing well academically and had no significant medical history prior to this illness.

On examination, he had tachycardia, tachypnoea and a fever of 102^°^F, however, oxygen saturation was maintained in room air. His was of thin build and appeared undernourished. According to the *World Health Organization*’s 2007 growth reference data for 5–19 years of age, his Z-scores for height-for-age and weight-for-age were -2.13 and -3.70, respectively, with a BMI of 13.2 kg/m^2^ (Z-score of -2.03).

**Figure 1:**
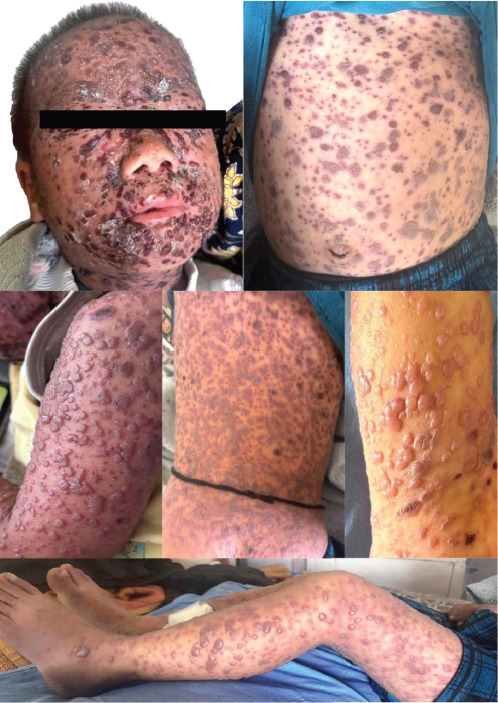
Monomorphic, tense vesicles with eczematous changes at presentation

Cutaneous examination revealed diffuse erythema and skin tenderness with extensive monomorphic, flat-topped, tense vesicles involving the head, trunk and bilateral extremities including the palms with scattered hemorrhagic vesicles and occasional pustules. Honey-colored crusting was evident over the peri-oral region and medial canthi (Figure 1).

**Figure 2:**
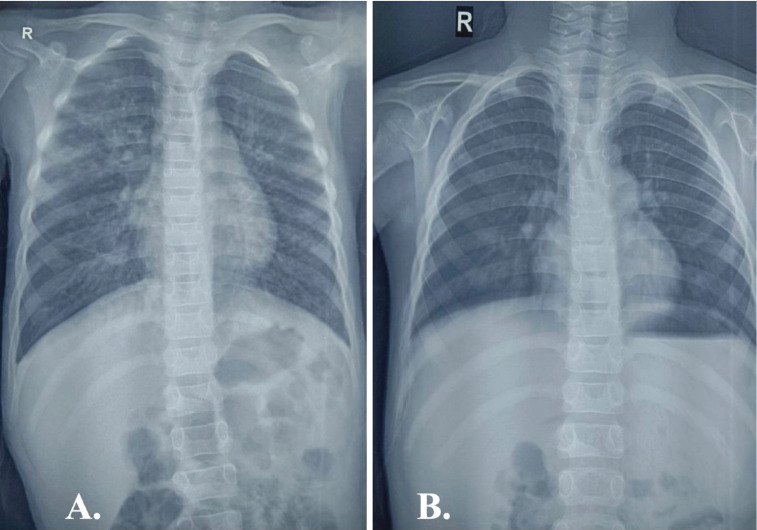
. (A.) CXR showing heterogeneous opacities involving the right upper lung zone at presentation (B.) Resolution of lung lesions post treatment completion

There was also evidence of bilateral conjunctival congestion and chemosis. On auscultation, coarse crepitations were evident predominantly over the right upper lung field. Heterogeneous opacities involving the right upper lung zone were noted in the chest radiograph (Figure 2).

**Figure 3:**
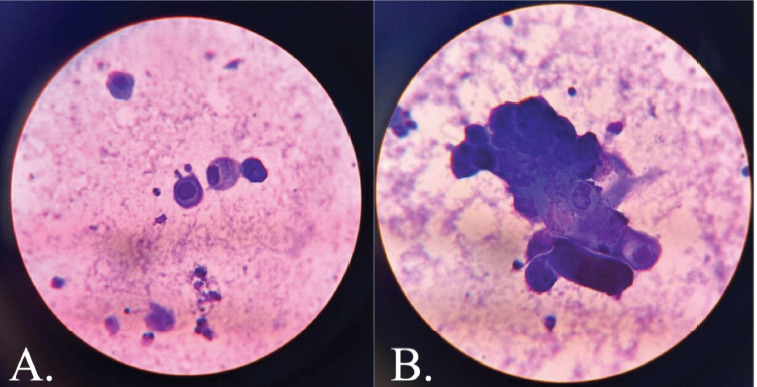
Tzanck smear showing (A.) acantholytic cells and (B.) multinucleate giant cells

**Figure 4:**
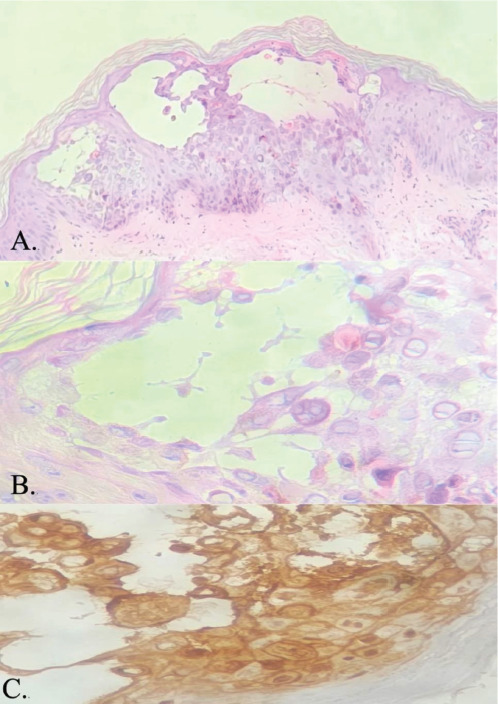
(A.) Intraepidermal blister with evidence of acantholysis (H&E, 100x) (B.) Viral infected cells showing characteristic nuclear changes of multinucleation, chromatin margination and molding (H&E, 400x) (C.) HSV-1 and HSV-2 immunohistochemistry positivity in the viral affected cells (IHC, 400x)

Tzanck smear revealed acantholytic cells and multinucleate giant cells (Figure 3). Skin biopsy findings were consistent with herpes infection showing intraepidermal blisters, with evidence of margination, multinucleation and nuclear moulding without any necrotic keratinocytes in the epidermis and direct immunofluorescence was negative for IgM/IgG/IgA/C3 (Figure 4). Viral serology showed positivity for HSV-1 and HSV-2 IgM and Tzanck smear revealed acantholytic cells and multinucleate giant cells (Figure 3). Skin biopsy findings were consistent with herpes infection showing intraepidermal blisters, with evidence of margination, multinucleation and nuclear molding without any necrotic keratinocytes in the epidermis, immunohistochemistry positivity for HSV-1 and HSV-2, and negative direct immunofluorescence for IgM/IgG/IgA/C3 (Figure 4). Viral serology showed positivity for HSV-1 and HSV-2 IgM and IgG.

**Figure 5:**
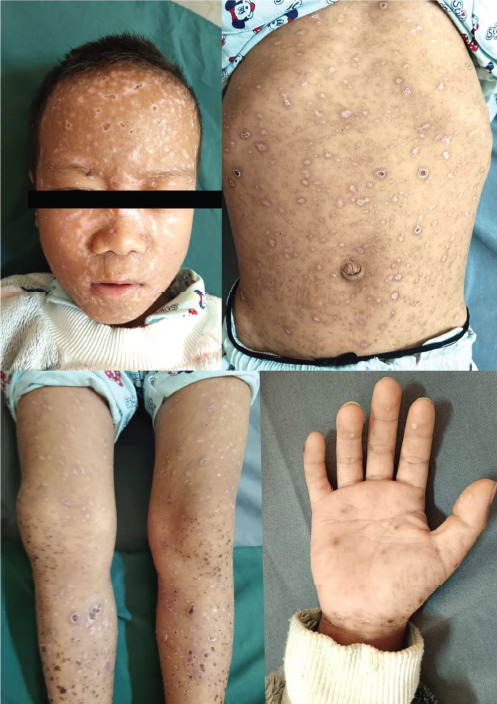
Areas of healing with hypopigmentation post antiviral therapy

Following the initial evaluation, the patient was shifted to pediatric ICU. Ultrasound-guided PICC line (a peripherally inserted central catheter) was placed due to challenging cannulation and sampling. His blood investigations revealed anemia, hepatic transaminitis, hyponatremia, and rise in acute phase reactants. He was started on a high dose intravenous amoxicillin-clavulanic acid at 25 mg/kg/dose thrice daily with hydration and correction of dyselectrolytemia. Subsequent to bedside evaluation of tzanck smear revealing multinucleate giant cells, the patient was started on intravenous acyclovir at 10 mg/kg/dose every 8 hours, and, after one day, his lesions started crusting. He was continued on intravenous antibiotics and antivirals for seven days, following which, it was converted to oral formulation for another seven days. Following the course of acyclovir, the lesions completely healed, leaving behind areas of hypopigmentation (Figure 5), and the patient was discharged with recommended dietary advice.

## Discussion

Kaposi varicelliform eruption (KVE) typically presents as painful umbilicated vesicles, which commonly localize to areas affected by a pre-existing skin condition [1–9]. However, in this case, while a few vesicles exhibited umbilication, the majority were tense, flat, and surrounded by an erythematous halo, and these lesions were disseminated throughout the body. The presence of diffuse erythema and tenderness may have contributed to the initial misdiagnosis as toxic epidermal necrolysis (TEN), leading to the administration of systemic steroids. This treatment likely exacerbated the patient’s condition, consistent with reports in the literature where immunosuppressive therapies have been associated with clinical deterioration (Table 1) [3–5,7].

**Table 1:** Various cases of KVE, their possible triggers and relevant findings

Reference	Cutaneous findings	Primary Dermatoses	Systemic comorbidities	Possible trigger	Tzanck Smear	Viral Serology	Histopathology
Zhong [3] (2024)	Disseminated monomorphic, umbilicated, vesiculopapular lesions with confluent areas of skin crusting and weeping over the entire face	Atopic dermatitis	SARS-CoV-2 infection	Dupilumab	Not done	Negative for EBV, CMV, VZV. HSV-1 positive by DNA. PCR	Not done
Sitaula [4] (2024)	Flaccid vesicles with haemorrhagic fluid, petechiae and purpura, erosions over the hard palate, thick crusts over the scalp	None	CKD with renal transplant	Tacrolimus, corticosteroids	Acantholytic cells, neutrophils, foamy macrophages	Not specified	Epidermis with intact basal layer with no vacuolar degeneration and superficial dermis showed minimal perivascular lymphocytic infiltrate
El-Masry [5] (2024)	Monomorphic vesicular skin eruption with few vesicles showing central umbilication, erythema, crusting	Varicella	None	NSAIDs	Not done	IgM for VZV	Epidermal vesicles with acantholytic cells, reticular degeneration, ballooning degeneration of keratinocytes
Martínez-Ortega [6] (2024)	Diffuse, grouped, tender papulovesicles, with erosions and crusting	Atopic dermatitis	None	None	Not done	IgM for HSV-1	Not done
Azmi [7] (2019)	Extensive umbilicated vesiculopustules with crusting	Varicella	CLD secondary to hepatitis C with liver transplant	Everolimus, prednisolone	Multinucleated giant cells with acantholysis and ground glass inclusions	Not done	Not done
Celtik [8] (2011)	Widespread vesiculopustular eruption with erosion and ulcers	Varicella	None	None	Acantholytic cells, multinucleate giant cells	IgM + IgG for HSV-1; IgM for VZV	Epidermal acantholysis, cellular ballooning, intraepidermal multinuclear giant cell. Immunohistochemical stains were positive for HSV
Shenoy [9] (2006)	Grouped vesicular lesions with umbilication and areas of chronic eczematous lesions	Atopic dermatitis	Bronchial asthma	None	Multinucleate giant cells, acantholytic cells,	Not done	Not done

Although serological tests and polymerase chain reaction (PCR) for varicella-zoster virus (VZV) could not be conducted due to limitations in resources and financial constraints, the clinical presentation of cutaneous lesions along with pulmonary involvement strongly suggested a varicella infection. The reported outbreak at the patient’s school further supported this diagnosis. Notably, tissue immunohistochemistry and serum IgM and IgG antibodies were positive for both HSV-1 and HSV-2, which is an unusual finding in our case, which may indicate herpes simplex virus reactivation on top of varicella. This reactivation could potentially be linked to the child’s chronic malnutrition, which may have compromised the immune response.

The tzanck smear provided a definitive diagnosis of herpesvirus infection, revealing characteristic features of acantholysis and multinucleate giant cells, consistent with findings in other reported cases [4,7–9]. This diagnostic method is particularly valuable in resource-limited settings, where viral serology and PCR may be inaccessible or expensive. In such contexts, the tzanck smear serves as a rapid and effective bedside diagnostic tool, enabling the timely initiation of treatment; as early intervention with acyclovir has been shown to yield significant clinical improvement in most cases of KVE, as demonstrated in our patient [2–9].

Histopathological examination proved to be another cost-effective and accessible diagnostic approach, playing a crucial role in excluding the possibility of a suspected drug reaction for which the patient was initially being treated for elsewhere.

## Conclusion

This case underscores the critical value of simple bedside tests, such as the tzanck smear, for rapid diagnosis of KVE and the timely initiation of treatment, facilitating prompt recovery. Additionally, it highlights the importance of histopathological evaluation in ambiguous cases to prevent mismanagement and ensure accurate diagnosis and appropriate care.
